# Integrating Diversity, Equity, and Inclusion into Preclinical, Clinical, and Public Health Mathematical Models

**DOI:** 10.1007/s11538-024-01282-4

**Published:** 2024-04-16

**Authors:** Justin Sheen, Lee Curtin, Stacey Finley, Anna Konstorum, Reginald McGee, Morgan Craig

**Affiliations:** 1 Department of Ecology and Evolutionary Biology, Princeton University, Princeton, USA; 2 Mathematical Neuro-Oncology Lab, Precision Neurotherapeutics Innovation Program, Mayo Clinic, Phoenix, AZ USA; 3 Department of Quantitative and Computational Biology, University of Southern California, Los Angeles, USA; 4 Alfred E. Mann Department of Biomedical Engineering, University of Southern California, Los Angeles, USA; 5 Mork Family Department of Chemical Engineering and Materials Science, University of Southern California, Los Angeles, USA; 6 University of Florida, Gainesville, FL USA; 7 Department of Mathematics and Computer Science, College of the Holy Cross, Worcester, USA; 8 Department of Mathematics and Statistics, Université de Montréal, Montréal, Canada; 9 Sainte-Justine University Hospital Azrieli Research Centre, Montréal, Canada

**Keywords:** Diversity, Equity, Inclusion, Mathematical modelling

## Abstract

Mathematical modelling applied to preclinical, clinical, and public health research is critical for our understanding of a multitude of biological principles. Biology is fundamentally heterogeneous, and mathematical modelling must meet the challenge of variability head on to ensure the principles of diversity, equity, and inclusion (DEI) are integrated into quantitative analyses. Here we provide a follow-up perspective on the DEI plenary session held at the 2023 Society for Mathematical Biology Annual Meeting to discuss key issues for the increased integration of DEI in mathematical modelling in biology.

## Introduction

Biomedical and public health modelling are used to describe a wide variety of biological processes and the interactions between them from the intracellular scale to the individual and population levels. Within and between each level, heterogeneity plays an important role in determining courses of action and outcomes, from pre-clinical to clinical research and for stakeholders and decision-makers in public health. Diversity, equity, and inclusion (DEI) are three fundamental pillars for the equal and fair participation of everyone, with a specific emphasis on traditionally underserved and marginalized groups. Though frequently discussed in the context of the researchers and clinicians leading and participating in a given study, there is also a need to apply a DEI lens to mathematical modelling approaches in preclinical, clinical and public health research (and beyond). Some factors to assess at the outset of a mathematical modelling study are the incorporation of data from a variety of sources and individuals, the appropriate use of modelling strategies and data analytic techniques that recover the heterogeneity observed in human populations, and the integration of populations that have generally and largely been excluded from biomedical and public health research. In these contexts, the attention paid to DEI has a direct impact on the availability and quality of the data required for modelling studies.

Representation, and the lack thereof, in clinical research has been recognized by the National Institutes of Health (NIH) in the United States as a critical component of successful clinical research (National Academies of Sciences et al. 2022). Furthermore, the NIH is mandated by law that women and members of racial and ethnic minority groups are included in all NIH-funded studies at a level appropriate for the study questions (Institutes and of Health 2022). Nevertheless, an NIH-commissioned report on diversity in clinical research at the United States Food and Drug Administration (FDA) and NIH between 2014 and 2020 showed an improvement in participation of women, but racial and ethnic disparities were generally maintained (National Academies of Sciences et al. 2022). In comparison to efforts in the context of clinical research, programs to systematically strengthen and evaluate diversity in preclinical research have historically received less attention. Initiatives such as the National Human Genome Research Institute (NHGRI) Human Genome Reference Program (National Human Genome Research Institute 2023), whose goal is to generate reference genome sequences that represent “as much as possible” of human haplotype diversity, seek to address this gap. Moreover, scientific societies are starting to play an increasing role in encouraging and contributing to DEI data initiatives. For example, the American Association for Cancer Research (AACR) has taken a multi-pronged approach to cancer health equity, including hosting sessions related to these topics at its recent annual meetings, setting up a cancer registry (AACR Project GENIE) to improve data sharing from diverse populations, and hosting focused conferences and workshops on cancer health disparities for underserved populations (American Association for Cancer Research 2024).

In line with these efforts, the Society for Mathematical Biology (SMB) formed the Diversity, Equity, and Inclusion Committee in 2020 as part of SMB’s “*ongoing commitment to cultivating an inclusive and equitable society*” (Society for Mathematical Biology 2023). As part of its activities, the DEI Committee has been organizing panel discussions, workshops, and minisymposia at the SMB Annual Meetings, as well as a virtual workshop in 2021. During the 2023 meeting held in Columbus, Ohio, USA and hosted by the Ohio State University, the DEI committee held a plenary session that included presentations and a panel discussion focused on integration of DEI principles in preclinical, clinical, and public health modelling research. To build upon this event, this paper discusses the issues and importance of DEI across biomedical and public health research from the perspective of mathematical biologists.

## DEI in the Biomedical and Public Health Modelling Context

The drug development pipeline, beginning with preclinical research in its various forms, is lengthy and costly. High prices associated with new drugs are related to attrition during the various phases required to develop a new drug. For example, it is estimated that it costs $1,943 million USD to bring new cell or gene therapies (Sabatini and Chalmers 2023) to the market, and recent estimates put the median cost of developing a new xenobiotic in any therapeutic area to be around $1,559.1 million USD (Wouters et al. 2020). There are a multitude of reasons new molecules may fail along the pipeline; these may include the omission of diverse cell lines (e.g., donors from both sexes, donors from different genetic ancestry, etc.) and animals (Flórez-Vargas et al. 2016) during the preclinical testing phase. From the outset, mechanistic models can be used to generate plausible mechanisms of disease and identify putative drug targets. Further, mathematical modelling can expand preclinical work through extrapolation to unconsidered variables and the hypothesis generation/testing paradigm to identify further experiments and research strategies. There is also a need to better understand new experimental systems used for preclinical research.

Advances in organoids and 3D bioprinting require an improved comprehension of their effects of the model system itself on outcomes. By considering diversity in various facets—from biobanks to cells to organoid systems (Co et al. 2023) to animals—preclinical mathematical models can be used to improve the success of candidate molecules and increase the successful transition into clinical trials. Historically, clinical trials, especially early-phase, have been run in a homogenous group of individuals (Merkatz 1998) (typically younger, Caucasian men) without the integration of gender, racial, ethnic, age, and other forms of diversity (Corneli et al. 2023). Increasing awareness of this reality has intensified calls for greater inclusions of human diversity within trials (Corneli et al. 2023; Washington et al. 2022). Recent advances in virtual clinical trial simulation and systems-level modelling have demonstrated the utility of mathematical and computational modelling for predicting different dosing schedule requirements needed in different populations. This is critical for reducing disparities in drug effects across different groups and makeups, and for getting necessary drugs to those who need them.

The integration of DEI is also called for to address health disparities in public health research. This was particularly highlighted throughout the COVID-19 pandemic, during which populational subgroups were at higher risk of contracting SARS-CoV-2 and of experiencing poorer outcomes for many reasons, including lack of access to care, more precarious working situations, more dense households, etc. For example, urban socioeconomic disparities in eviction rates as well as the ability to social distance are crucial to fully understand disease transmission in cities. Predictions from an epidemiological network model showed that not only do these disparities lead to consequent disparities in infection burden depending on socioeconomic status, but that in general, the infection burden for a city will increase (Nande et al. 2021) due to both evictions and higher contact rates, and a lessened ability to social distance. A failure to integrate data on historically underrepresented groups would mean missing a crucial characteristic of transmission for any urban setting. Capturing the interplay between disparities and infection transmission will always require more complicated models, requiring more complex theory, data, and simulation. Although challenging, the insights gained from such approaches can be more relevant for our interconnected and heterogeneous world.

## Challenges for the Field of Mathematical Biology: Where Do We Go from Here?

It is an opportune time to discuss what mathematical biologists and modellers, whether they apply their efforts towards (pre-)clinical and public health problems or beyond, can do to better respond to and include DEI in their models. Technical aspects could include departing from seeking a single parameterization to considering a range of parameterizations for a model to encompass observed diversity (Craig et al. 2023), modifications of model structure to better represent differences between individuals/groups of individuals and using parameter estimation techniques that can account for heterogeneity (e.g., nonlinear mixed effects models, etc.). There is also a need to continue to develop methodologies that incorporate complexity in the form of between-subject variability (Nande et al. 2021; Brady-Nicholls et al. 2021; Ojwang et al. 2024).

At the outset of a project, it is critical that modellers reflect on the limits of their data, and whether certain groups may have been excluded from data collection, as discussed above in the case of clinical trials. A model could, for example, be used for hypothesis generation to predict potential outcomes in groups not included during data collection. As models become more complex when more groups are included (Sandoval-Olascoaga et al. 2021), such approaches motivate increased data collection in all relevant areas of biological research. In the context of preclinical, clinical, and public health research, this underlines the importance of working closely with experimentalists, clinicians, and field researchers who collect and generate the data used within our models, as well as who develop the theory that our model assumptions are based upon.

## Conclusion

As mathematical modelling becomes increasingly integrated within biological disciplines, including preclinical and clinical research, and public health, it is critical to assess its contribution to the fundamental issues of diversity, equity, and inclusion. In addition to advancing the technical and methodological aspects of mathematical biology, the Society for Mathematical Biology and the DEI Committee are dedicated to fostering an inclusive SMB through multiple events at the annual meeting and by engaging in conversations with the mathematical biology community at all stages.

Ultimately, taking diversity, equity, and inclusion into account during the modelling process will fuel new lines of research and ensure the applicability and relevancy of mathematical models and their predictions to all. The consideration of DEI in modelling demonstrates the broader importance of engaging with traditionally underserved and underrepresented groups in mathematical biology and research at large.

## Data Availability

This manuscript contains no associated data.
